# Antifungal and antibacterial activity and chemical composition of polar and non-polar extracts of *Athrixia phylicoides* determined using bioautography and HPLC

**DOI:** 10.1186/1472-6882-13-356

**Published:** 2013-12-13

**Authors:** Lyndy Joy McGaw, Victor Patrick Bagla, Paul Anton Steenkamp, Gerda Fouche, Jana Olivier, Jacobus Nicolaas Eloff, Martin Steven Myer

**Affiliations:** 1 Department of Paraclinical Sciences, Phytomedicine Programme, Faculty of Veterinary Science, University of Pretoria, Private Bag X04, Onderstepoort 0110, South Africa; 2 Present address: Department of Biochemistry, Microbiology and Biotechnology, University of Limpopo, Private Bag X1106, Sovenga 0727, South Africa; 3 Biosciences, Council for Scientific and Industrial Research, PO Box 395, Pretoria, Gauteng 0001, South Africa; 4 Department of Biochemistry, University of Johannesburg, Auckland Park 2006, South Africa; 5 Department of Geography, Geoinformatics and Meteorology, University of Pretoria, Private Bag X20, Hatfield 0028, South Africa

**Keywords:** Antibacterial, Antifungal, Asteraceae, *Athrixia phylicoides*, Bioautography, HPLC, Zulu tea

## Abstract

**Background:**

*Athrixia phylicoides* DC. (Asteraceae) is used medicinally in South Africa to treat a plethora of ailments, including heart problems, diabetes, diarrhoea, sores and infected wounds. It is also prepared in the form of a tea (hot decoction) taken as a refreshing, pleasant-tasting beverage with commercialization potential.

**Methods:**

Extracts of the dried ground aerial parts were prepared using organic solvents (diethyl ether, dichloromethane/methanol, ethyl acetate and ethanol) and water. These extracts were subjected to HPLC, TLC and bioautography analysis with the aim of linking a range of peaks visualized in HPLC chromatography profiles to antibacterial and antifungal activity of the same extracts.

**Results:**

HPLC revealed a group of compounds extracted by more than one solvent. Compounds identified include inositol, caffeic acid, quercetin, kaempferol, apigenin, hymenoxin and oleanolic acid. The organic extracts displayed similar TLC profiles, and bioautography indicated approximately five antibacterial compounds, but only two antifungal compounds in these extracts. Bioautography indicated that cold water extracted the least antimicrobial compounds.

**Conclusions:**

Several previously unknown compounds were identified in *Athrixia phylicoides* extracts, and bioautography indicated a number of antibacterial and antifungal compounds. There were notable differences in chemical composition and bioactivity between the organic and aqueous extracts. Further research is necessary to fully characterize the active components of the extracts.

## Background

**Figure 1:**
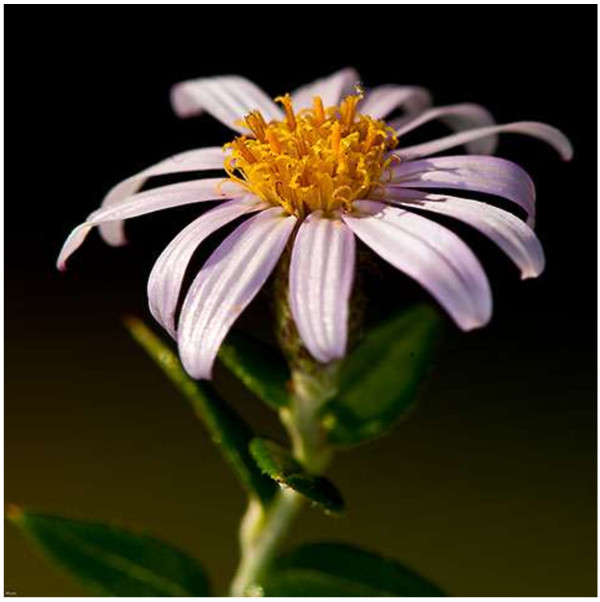
***Athrixia phylicoides *** **(photograph: Alvaro Viljoen), voucher number PRE 592582.0.**

Of the fourteen species of the genus *Athrixia,* nine are found in South Africa [1], including *Athrixia phylicoides* DC. (Asteraceae), which grows in mountainous and grassland areas of the eastern parts of South Africa [2]. *Athrixia phylicoides*, commonly known as bush tea or Zulu tea, is an aromatic leafy shrub with purple daisy-like flowers (Figure 1), growing mainly in grassland and forest margin scrub regions. Following the commercial successes of the South African rooibos (*Aspalathus linearis*) and honeybush (*Cyclopia* spp.) teas, research into the potential commercialisation of *Athrixia* Zulu tea is being encouraged [3]. A recent survey of rural and urban dwellers in northern parts of South Africa revealed an active informal trade in the herb, used as a tea and for preparation of aromatic brooms from twigs stripped of leaves [4]. Rooibos and honeybush tea were originally drunk medicinally and then increasingly as a refreshing beverage, and it is envisioned that *Athrixia* tea may follow a similar path as an antioxidant-rich, caffeine-free tea [5-9]. In a review of research done on *Athrixia phylicoides*[3], it was concluded that in terms of quality attributes and current use, Zulu tea has promising potential for development as a commercial product.

Decoctions and infusions prepared from the leaves and twigs of *A. phylicoides* are widely used by rural people to treat a variety of ailments, including sores, boils, acne, infected wounds [10], hypertension, circulation and heart problems, diabetes, diarrhoea, vomiting and skin conditions [4]. Leaf and root extracts are used as an anthelmintic [11] and as a remedy for cough [12], while root decoctions are used as a purgative [12]. A survey conducted in the Limpopo Province of South Africa revealed that Zulu tea is used to treat headaches, stomachache, influenza and leg wounds [13]. It is also used to cleanse the womb, kidney and veins and to purify blood [13]. The tea is purported to have stimulant and aphrodisiac properties [10,13,14]. *A. phylicoides* leaves and twigs are also prepared as a hot decoction and drunk as a pleasant-tasting, non-medicinal tea by indigenous South Africans. The preparation usually involves initially boiling a handful of broken leaves and twigs in approximately 1.5 L of water for 5 min, after which the decoction is strained and served, usually without milk, and with sugar added to taste [4]. According to a recent survey, more rural and urban respondents use the plant as a tea rather than for medicinal purposes [4].

Biological activity studies on *Athrixia phylicoides* have concentrated on determining the antioxidant potential of the plant material as affected by time of year and other factors [5-9,15,16]. Antibacterial activity of the ethanol extract has been studied [6], together with anti-inflammatory, antimicrobial and antimalarial properties of the methanol extract and essential oil [9]. A hot water extract of *A. phylicoides* twigs and leaves contained the phenolic compounds 6-hydroxyluteolin-7-O-glucoside, chlorogenic acid, protocatechuic acid, a di-caffeoylquinic acid and a methoxy-flavonol derivative [17]. This extract stimulated *in vitro* glucose uptake and metabolism, suggesting that consumption of the tea could potentially ameliorate metabolic disorders related to obesity and Type 2 diabetes mellitus [17].

Compounds isolated from *A. phylicoides* include chlorogenic acid, 1,3-dicaffeoylquinic acid, several hydroxycinnamic acid derivatives, including dicaffeoyl quinic acids, and an unidentified flavone–hexose as well as 6-hydroxyluteolin-7-O-B-glucoside and quercetagetin-7-O-B-glucoside [15]. Other researchers [18] identified germacrene D, linoleic acid and *p*-hydroxyphenylpropan-3-ol-coumarate, while a novel flavonol derivative, 5-hydroxy-6,7,8,3′,4′,5′-hexamethoxyflavon-3-ol has also been identified from the leaves of *Athrixia phylicoides*[3,19]*.* The same compound, as well as three more known flavonoids, namely 3-O-dimethyldigicitrin, 5,6,7,8,3′,4′-hexamethoxyflavone and quercetin, has been isolated from the ethanol extract of *A. phylicoides* aerial parts [7]. Three polymethoxylated flavones, quercetin-3′–O-glucoside, as well as a methoxylated derivative, two dicaffeoyl quinic acids and one coumaric acid ester have also been identified in the methanolic extract of Zulu bush tea [20]. In a similar study comparing with authentic standards, protocatechuic, *p*-coumaric, caffeic and chlorogenic acids from *Athrixia phylicoides* extracts were identified [5]. In addition, a section of their spectra corresponding to dicaffeoylquinic acids, were also tentatively identified. However, Zulu tea leaves in another study were shown to be devoid of caffeine or pyrrolizidine alkaloids [8]. It has been postulated that the chemical composition of Zulu tea plants is affected by seasonal nitrogen, phosphorous and potassium nutrition [21-25], thus helping to explain the variable findings between researchers.

A subchronic toxicity study of the water extract of *A. phylicoides* was undertaken in rats [26]. No morbidity or mortality was noted, and the biochemical parameters and histopathology of internal organs showed no indication of toxicity. Regarding *in vitro* studies, it has previously been found that although the water extract of *A. phylicoides* was not toxic to Vero cells at the highest concentration tested of 1 mg/ml [8], the ethanol extract was relatively more toxic with IC_50_ values ranging between 100 and 400 μg/ml against Vero cells and brine shrimp larvae [7,8]. These IC_50_ values do not represent significant toxicity. It was however previously reported that four flavonoids isolated from an *A. phylicoides* ethanol extract were more cytotoxic to Vero cells, with IC_50_ values as low as 28 μg/ml [7].

Based on traditional use, existing phytochemical knowledge and lack of toxicity, it was decided to screen the plant for antibacterial and antifungal activity using the technique of bioautography and to determine which phytochemicals could be extracted with different solvents using HPLC. Bioautography is a useful bioassay for localizing antibacterial or antifungal activity of plant extracts after separation of the constituent compounds on a thin layer chromatography (TLC) plate. Agar overlay methods have been used in the past where an agar medium seeded with test organism is applied directly to the TLC plate [27,28]. However, adequate diffusion of active compounds through an aqueous agar matrix may be problematic, particularly in the case of non-polar extracts [29]. In the direct bioautography technique, plant extracts are separated using an appropriate solvent system, the eluting solvent is dried from the TLC plates and then a fine suspension of actively growing bacterial or fungal culture is sprayed on the TLC plate [30]. This method has been modified for use with *Candida albicans* and *Cryptococcus neoformans*[31]. Bioautography allows the determination of the number of active compounds in different extracts or fractions, as well as a comparison of inhibition of different micro-organisms by compounds at the same R_f_ values on TLC plates. The occurrence of the same active compounds in different extracts can also be deduced. The strength of the inhibition zone additionally permits comparison to a degree of the relative activities (and hence possibly the relative quantities) of different inhibitory compounds in a plant extract or fraction.

In a recent review on South African herbal teas [32], it was concluded that future research on tea species including *Athrixia phylicoides* should be directed towards more comprehensive chemical characterisation of extracts, and identification of marker compounds for extract standardisation and quality control. There is little information available on the potential health benefits with regard to antibacterial and antifungal compounds present in *A. phylicoides*, although indications for its traditional use point to possible antimicrobial efficacy. The chemical composition of *Athrixia phylicoides* has also not been thoroughly studied. The present study was undertaken to address these deficits in knowledge of *Athrixia phylicoides* prior to further work on commercialization. The differences between chemicals extracted using organic and aqueous solvents were investigated using HPLC and bioautography analysis methods. Using HPLC, the complexity of the plant extracts could be determined, and an indication of the types of chemical compounds present could be provided. In the bioautography analysis, the presence and number of antibacterial and antifungal compounds could be localized on a chromatogram and compared between the extracts prepared using solvents of different polarities.

## Methods

### Plant material

*Athrixia* plant material (aerial parts) was collected and subsequently cut into small pieces and dried in an oven at 30–60°C. A voucher specimen was deposited in the herbarium of the South African National Botanical Institute (SANBI) in Pretoria (voucher number PRE 592582.0; GENSPEC number 308615). The identity of the plant material was verified by qualified taxonomists based at SANBI, a national reference centre. Dried material was ground to a coarse powder using a hammer mill and stored at ambient temperature prior to extraction.

### Preparation of extracts

The powdered plant material (20 g quantities) was extracted using a variety of solvents spanning a range of polarity, namely diethyl ether (DEE; yield 0.95 g, 4.8%) (extract 1), dichloromethane/methanol (DCM/MeOH = 1:1; yield 1.08 g, 5.4%) (extract 2), ethyl acetate (EtOAc; yield 1.10 g, 5.5%) (extract 3), ethanol (EtOH; yield 0.88 g, 4.4%) (extract 4) and room temperature water (H_2_0; yield 4.40 g, 22.0%) (extract 5) in a standard extraction technique. The powdered material was shaken for 30 min with the extracting solvent (1:10 ratio) and filtered before being dried and stored at 4°C until analysis. Saturated solutions of each extract were prepared by adding an aliquot of the dried extracts to the corresponding solvent, sonicating the samples using an Integral Systems Ultrasonic Bath (UMC 20+ Heater, 50 Hz) for 30 minutes and filtering the resulting solutions through a 0.45 μm syringe filter. The filtered samples were then analyzed according to a variation of the HPLC method developed by Kamatou and co-workers [33], as well as by thin layer chromatography (TLC) and bioautography [30].

### HPLC analysis

#### Instrumental conditions for HPLC analysis

The analysis was done on a Waters Thermabeam (TMD) HPLC system comprising a 2695 Solvent Delivery System, a 2996 PDA detector, column heater and Thermabeam (TMD) EIMS detector. The PDA (photodiode array, non-destructive) detector established the light absorbance spectra from visible and UV wavelengths of each detected compound, while the TMD detector (destructive method) was placed after the PDA in order to generate the corresponding mass spectra. Chromatographic separation was performed on a Phenomenex Gemini C18 column, 250 × 2 mm (5 μm 125A) maintained at 40°C. The starting eluent consisted of water containing 10 mM formic acid (FA) (Phase A) and acetonitrile (ACN) (Phase B) in a 90:10 ratio at 0.2 ml/min. The initial gradient table is summarized in Table 1 (screening method 02) but several other methods were tested. The extracts were first evaluated on the Gemini column and thereafter re-analyzed on a Waters Xbridge C18 column 150 × 2.1 mm (3.5 μm 110A) but replacing the acetonitrile with methanol (Phase C) (screening method 03, Table 2). Both columns were equipped with guard columns of identical packing material and a mechanical filter was inserted to trap any particulate matter or possible precipitates.

**Table 1:** Gradient conditions of screening method 02 on the Waters 2695

**Time**	**Flow**	**%Phase A**^**1**^	**%Phase B**	**Curve**
0.0	0.20	90	10	6
1.0	0.20	90	10	6
40.0	0.20	0	100	6
45.0	0.20	0	100	6
50.0	0.20	90	10	3
58.0	0.20	90	10	6
59.0	0.20	90	10	6

^1^Phase A = water with 0.1% formic acid, Phase B = acetonitrile.

**Table 2:** Gradient conditions of screening method 03 on the Waters 2695

**Time**	**Flow**	**%Phase C**^**1**^	**%Phase A**	**Curve**
0.0	0.20	10	90	6
1.0	0.20	10	90	6
40.0	0.20	100	0	6
45.0	0.30	100	0	6
50.0	0.20	10	90	3
58.0	0.20	10	90	6
59.0	0.20	10	90	6
78.0	0.20	100	0	6

^1^Phase C = methanol, Phase A = water with 0.1% formic acid.

The TMD was operated in positive scan mode (50 – 650 amu) with a gain of 10, the nebulizer temperature was set at 70°C, the expansion region temperature at 80°C and the source temperature at 225°C. The total volume of post-column eluent was sent to the PDA and TMD detectors. The TMD detector was tuned every day prior to starting analysis and caffeine was injected as a test compound to ensure functionality of the total system.

### TLC and bioautography

#### Thin layer chromatography (TLC) and phytochemical analysis

The dried extracts were dissolved in ethanol to a concentration of 10 mg/ml. Chemical constituents of the extracts (100 μg) were separated using aluminium-backed TLC plates (Merck, silica gel 60 F_254_). TLC plates were prepared in triplicate and each TLC plate was developed using one of three eluent systems as follows: ethyl acetate:methanol:water (40:5.4:5) [EMW] (polar, neutral); chloroform:ethyl acetate:formic acid (5:4:1) [CEF] (intermediate polarity, acidic); benzene:ethanol:ammonium hydroxide (90:10:1) [BEA] (non-polar, basic) [34]. To completely remove traces of the eluting solvents, the TLC plates were dried under a stream of cold air until there was no solvent smell remaining. The TLC plates were then sprayed with vanillin-sulphuric acid spray reagent (0.1 g vanillin:28 ml methanol:1 ml sulphuric acid) and heated at 110°C until optimal colour development [35].

#### Bacterial and fungal cultures

The bacterial species used were the Gram-negative *Escherichia coli* (ATCC 25922) and *Pseudomonas aeruginosa* (ATCC 27853) and the Gram-positive *Staphylococcus aureus* (ATCC 29213) and *Enterococcus faecalis* (ATCC 29212). Fungal species included clinical isolates of *Candida albicans* (from a Gouldian finch) and *Cryptococcus neoformans* (isolated from a cheetah), which were obtained from the culture collection of the Department of Veterinary Tropical Diseases, University of Pretoria. Bacteria were maintained on Müller-Hinton (MH) agar (Merck) at 4°C and were cultured in MH broth (Fluka) at 37°C before use in the bioassays. Fungi were maintained on Sabouraud Dextrose (SD) agar (Fluka) at 4°C and were cultured in SD broth (Merck) at 35°C and incubated overnight prior to conducting bioautography and microdilution assays.

#### Bioautography assay

For each bacterial and fungal test organism used, a set of three TLC plates was prepared as for the TLC analysis above, and allowed to dry completely. The TLC plates were then sprayed with a concentrated suspension of bacterial cells or fungal conidia grown overnight in appropriate broth [30,31]*.* The TLC plates sprayed with bacterial or fungal suspensions were incubated overnight at 37˚C or 35˚C respectively under 100% relative humidity (in a sealed plastic container) to allow growth of the micro-organism on the plates. The bioautograms were then sprayed with an aqueous solution of 2 mg/ml *p*-iodonitrotetrazolium violet (INT, Sigma) and incubated at 37˚C or 35˚C respectively for approximately four hours until appearance of a red colour showed growth of the microorganisms. Following incubation, the development of clear zones against a red background indicated inhibition of fungal or bacterial growth by bioactive compounds separated on the TLC plates.

## Results

### HPLC analysis

After preliminary trials, optimization of the chromatographic procedure involved 5 extracts (1 – 5) and two analytical profiles, the first using methanol and a Gemini C18 column (Table 1) and the other using acetonitrile and a Waters Xbridge C18 column (Table 2). In the HPLC analysis of the extracts, the better separation was attained by using the gradient profile “screening method 02” (Table 1). The Waters Xbridge C18 column improved the peak shape of the compounds from those obtained on the Gemini column. The replacement of acetonitrile with methanol improved the resolution of the compounds although co-elution was not completely removed using this “screening method 03” (Table 2).

**Figure 2:**
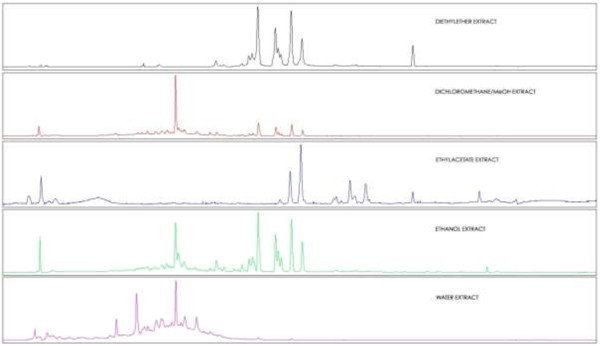
**Stacked UV chromatograms (200 – 600 nm) of the five extracts (DEE, DCM/MeOH, EtOAc, EtOH and H**_**2**_**O from top to bottom) obtained with screening method 02.**

The stacked chromatograms (chromatograms prepared by overlaying chromatographic profiles of extracts separated after several injections) show clearly that no one extracting solvent extracted all phytochemicals seen in the UV chromatograms (Figure 2). In Figure 2 the intensities of the various chromatograms were individually normalised to the largest detected peak.

**Figure 3:**
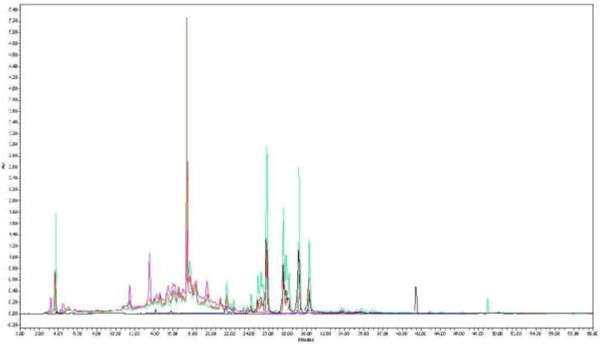
**Overlaid UV chromatograms of the five extracts obtained with screening method 02 (Gemini column).** Colours of chromatograms: black = DEE extract, red = DCM/MeOH extract, blue = EtOAc extract, green = EtOH extract, pink = H_2_O extract.

**Figure 4:**
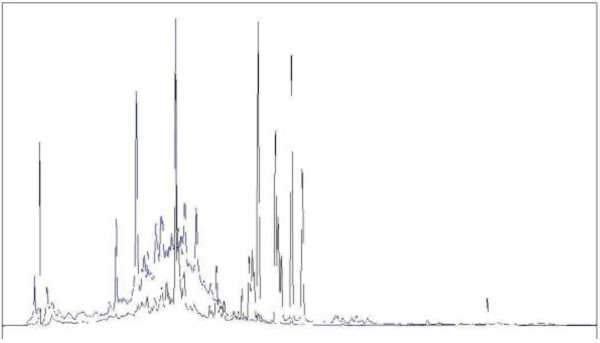
**MaxPlot UV chromatograms of the EtOH (black) and H**_**2**_**O (blue) extracts (screening method 02).**

The chromatograms shown in Figure 2 were overlaid by means of a software facilitated overlay procedure to produce Figure 3. The intensities of the overlaid chromatograms in Figure 3 were then normalized to the large, saturated peak detected in the DCM/MeOH extract (extract 2). Figure 3 clearly shows that extracts 1 to 5 contain phytochemicals that were extracted by more than one of the solvent systems used. Except for a few compounds unique to the H_2_O (extract 5) and EtOAc (extract 3) extracts, the majority of compounds were successfully extracted with EtOH (extract 4). The concentration of the extracted compounds in the EtOH extract was higher than for the other extracting solvents except for the one large peak observed in the DCM/MeOH extract (extract 2, red chromatogram). The compounds of the EtOH extract displayed similar chromatographic behaviour by co-eluting rather than separating into individual compounds, which is an indication of structural similarities, as well as closely related polarities. A comparison of the different peaks found in the EtOH and H_2_O extracts is shown in Figure 4, where the chromatograms resulting from the two extracts have been overlaid.

Masses of interest in the EtOH extract were sequentially extracted and searched against the NIST mass spectral library. None of the intact glycosides could be detected which was not surprising, as these compounds are labile and not easily detected by classical EI ionisation techniques. A few compounds were tentatively identified, mainly in the EtOH extract, and are listed in Table 3, together with the extracts in which they were detected.

**Table 3:** **Identified compounds in *Athrixia phylicoides *** **extracts prepared using different solvents**

**COMPOUND**	**FORMULA**	**MASS**	**DEE**	**DCM/MeOH**	**EtOAc**	**EtOH**	**H**_**2**_**0**
Inositol	C_6_H_12_O_6_	180	X^1^	X	X	√	√
Caffeic acid	C_9_H_8_O_4_	180	X	X	X	√	√
Quercetin	C_15_H_10_O_7_	302	X	X	X	√	X
Kaempferol	C_15_H_10_O_6_	286	√	√	X	√	X
Apigenin	C_15_H_10_O_5_	270	X	√	X	√	X
Hymenoxin	C_19_H_48_O_8_	374	√	√	√	√	X
Oleanolic acid	C_30_H_48_O_3_	456	X	X	X	√	X
7,7′-Dihydroxy-8,8′-dimethoxy-3,3′-dimethyl-2,2′-binaphthalene-1,1′,4,4′-tetrone	C_24_H_18_O_8_	434	√	√	√	√	√
Scaposin	C_19_H_18_O_9_	390	√	√	X	√	√

^1^X = absent, √ = present.

### TLC and bioautography analysis

**Figure 5:**
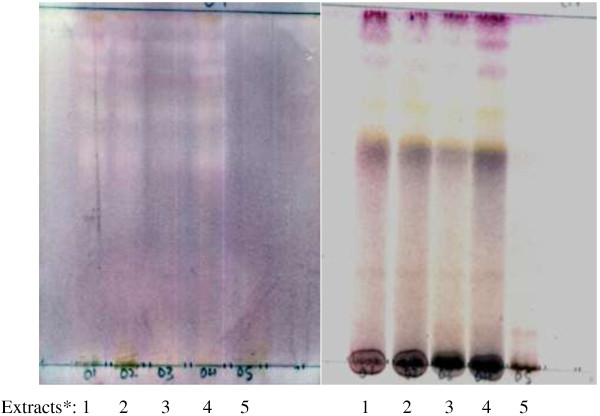
**TLC plates eluted in CEF solvent system (chloroform:ethyl acetate:formic acid = 5:4:1) and sprayed with *Enterococcus faecalis *(left) and vanillin-sulphuric acid spray reagent (right).** The solvent fronts and origins are marked with pencil lines at the top and bottom of the TLC plates respectively. *Extracts: 1 = DEE extract, 2 = DCM/MeOH extract, 3 = EtOAc extract, 4 = EtOH extract, 5 = H_2_O extract.

TLC analysis revealed that extracts 1, 2, 3 and 4 (DEE, DCM/MeOH, EtOAc and EtOH extracts respectively) had very similar phytochemical profiles, although extracts 1 and 4 appeared the most alike, while extracts 2 and 3 contained lower quantities of certain compounds as evidenced by lighter bands on the chromatograms. Extract 5 (the water extract) was clearly distinct from the other extracts, showing far fewer compounds when TLC plates were sprayed with the vanillin-sulphuric acid spray reagent (Figure 5).

The CEF solvent system was the most efficient at separating the constituent compounds of the extracts (1 – 5) of all the solvent systems used (EMW, CEF and BEA) as shown by a greater number of separated bands for each extract. With the polar EMW solvent system, all the visible active zones separated poorly and appeared close to the solvent front of the TLC chromatogram, indicating that the active compounds were relatively non-polar (results not shown). The polar BEA solvent system was also less efficient than the CEF, with active zones appearing at Rf values below 0.5 in almost all cases (data not shown).

#### Bioactivity

Bioactivity against the Gram-positive *E. faecalis* in the CEF eluting solvent system (for extracts 1 – 4) was detected at the bands with R_f_ = 0.59 and 0.71, correlating to brownish-yellow compounds, and also at bands of R_f_ = 0.84 and 0.89, corresponding to reddish-pink compounds suggestive of flavanol-type compounds in the reference TLC plate (Table 4) sprayed with vanillin spray reagent. Vanillin is an aromatic aldehyde which reacts with the metasubstituted ring of flavanols to yield a red adduct [36]. No zones of inhibition against *E. faecalis* could be seen in extract 5. An example of a TLC plate sprayed with *E. faecalis* as test organism, and a reference TLC plate sprayed with vanillin-sulphuric acid spray reagent, is shown in Figure 5.

**Table 4:** **R**_**f **_**values of zones of bacterial and fungal growth inhibition by *Athrixia *** **extracts when separated by TLC with CEF as eluent**

**R**_**f **_**value**		***E. coli***^***a***^		***E. faecalis***		***S. aureus***		***C. albicans***		***C. neoformans***	
	Extracts^b^:	1 - 4	5	1 - 4	5	1 - 4	5	1 - 4	5	1 - 4	5
0.58^1^	Yellow-brown	−^4^	-	-	-	√	-	-	-	-	-
0.59^1^	Yellow-brown	-	-	√	-	-	-	√	√	√	-
0.64	Yellow-brown	√	-	-	-	√	-	-	-	-	-
0.70^2^	Yellow-brown	√	-	-	-	-	-	-	-	√	-
0.71^2^	Yellow-brown	-	-	√	-	-	-	-	-	-	-
0.72^2^	Yellow-brown	-	-	-	-	√	-	√	-	-	-
0.84	Pink	√	-	√	-	-	-	-	-	-	-
0.89^3^	Pink	-	-	√	-	√	-	-	-	-	-
0.91^3^	Pink	√	√	-	-	-	-	-	-	-	-

^1,2,3^compounds with the same superscript are likely to be the same compound, after comparison with the reference TLC plate sprayed with vanillin-sulphuric acid.

^4^**-** = active compound not present, **√** = active compound present.

^a^*E. coli = Escherichia coli, E. faecalis = Enterococcus faecalis, S. aureus = Staphylococcus aureus, C. albicans = Candida albicans, C. neoformans = Cryptococcus neoformans.*

^b^Extracts: 1 = diethyl ether extract, 2 = DCM/MeOH extract, 3 = EtOAc extract, 4 = EtOH extract, 5 = H_2_O extract.

Pronounced antibacterial activity against the Gram-positive *S. aureus* was noted at the bands at R_f_ = 0.58, 0.64, 0.72 and 0.89 in extract 4 when using CEF as the eluting system (Table 3). These compounds (yellow-brown and pink) were also visible in extracts 1, 2 and 3, although they were not as distinct. No clear zones of inhibition against *S. aureus* could be seen in extract 5.

Using CEF as eluent, several bioactive zones on the TLC plate could be distinguished against *E. coli* (Gram-negative) in extracts 1 to 4, at R_f_ = 0.64, 0.70, 0.84 and 0.91. The first two zones (R_f_ = 0.64 and 0.70) appeared to correlate with brownish-yellow spots while the other two active compounds correlated with pink bands on the vanillin-sprayed reference plate. The active zone at R_f_ = 0.91 was also detected in the extract 5. It should be noted that the “-” signs in Table 4 refer to lack of activity of individual compounds eluting on the TLC plates at those particular Rf values, and do not indicate lack of activity of the entire extract, as compounds eluting at other Rf values may show activity. No activity was detected against the Gram-negative *P. aeruginosa* (results not shown).

Antifungal activity after eluting in the CEF solvent system against *Candida albicans* was noted with the zone at R_f_ = 0.59 in all five extracts (less distinct in extract 5), and a very faint possible zone of activity occurred at R_f_ = 0.72 in extracts 1 to 4. These zones both correlated with brownish-yellow bands on the reference TLC plate. Antifungal activity (also in the CEF solvent system) against *Cryptococcus neoformans* was noted with the band at R_f_ = 0.59 and 0.70 in extracts 1–4. These bands were brownish-yellow in the reference TLC plate. It was difficult to distinguish whether there was any activity against *Cryptococcus neoformans* in extract 5.

In summary, it appears that compounds at the same R_f_ values of approximately 0.58 (brownish-yellow), 0.72 (brownish-yellow) and 0.89 (pink) were active against the Gram-positive test organisms *E. faecalis* and *S. aureus*. A brownish-yellow compound at R_f_ = 0.64 was active against *S. aureus* but not against *E. faecalis*, and a pink compound at R_f_ = 0.84 was active against *E. faecalis* but not *S. aureus*. No zones of inhibition against Gram-positive bacteria were apparent in extract 5.

The same brownish-yellow compounds at R_f_ = 0.64 and 0.71 in extracts 1–4 that were active against the Gram-positive bacteria were also active against the Gram-negative *E. coli*. The pink band at R_f_ = 0.84 in extracts 1–4 was active against both *E. coli* and Gram-positive *E. faecalis*. A further pink band at approximately R_f_ = 0.90 was active against *E. coli* as well as both Gram-positive species, and this active zone was also seen in extract 5 against *E. coli*. No antibacterial activity was noted against the Gram-negative *P. aeruginosa* in the bioautography assays.

## Discussion

The traditional ways of preparing a herbal tea involve boiling plant material in water to yield a decoction or soaking plant material in freshly boiled water to produce an infusion. In addition, for some plants, in the hands of ‘more experienced’ traditional herbalists, a pre-layup process is carried out prior to producing the water infusion (J Olivier, personal communication). This pre-layup process often involves carrying plant material around on their heads under some kind of hat, with the resultant fermentation possibly following an ‘ethanol-type’ extraction process.

In this study, the ethanol extract produced a more complex mixture of compounds than the water extract, especially of intermediate polarity, as there were significantly more bands in the TLC chromatogram for the ethanol extract when compared to those visualized in the water extract (Figure 5). Although some observed peaks correlated between the water and ethanol extracts in the HPLC analysis (Figure 4), the two extracts differed significantly, which raises the question regarding the difference in herbal/medicinal potency, as well as toxicity, between the two extracts. The bioactivity of the organic solvent extracts (1 – 4) appears to have been similar and the toxicity of the extracts could be explored in future work. It was previously reported that ethanol extracts were more toxic than aqueous *A. phylicoides* extracts [8]. Many investigations have shown that aqueous extracts are less toxic than ethanol extracts [37-39]. Chemicals found in the ethanol extract in this study included quercetin, kaempferol, apigenin, hymenoxin and oleanolic acid (Table 3). The compounds quercetin [40], kaempferol [41], apigenin [42] and oleanolic acid [43] do not appear to be associated with toxicity but higher doses of flavonoids may have prooxidant activity and hence may be implicated in cytotoxicity [42]. However, the sesquiterpene lactone, hymenoxin, is potentially toxic [44,45], and the presence of this compound in the ethanol extract may contribute to the reported toxicity of this extract.

In another study [46], it was found that fermented water infusions of rooibos tea (*Aspalathus linearis*) were more effective in combating the growth of *Escherichia coli* than the unfermented rooibos extract. Zulu tea (*Athrixia phylicoides*) is not fermented before preparation, but this is an aspect that could be investigated in the future to provide different tastes for evaluation prior to potential commercialization, particularly if fermentation results in an improved taste or a different chemical composition which may enhance health benefits.

In earlier research, it was discovered that infusions and decoctions prepared following traditional methods and laboratory-prepared cold aqueous extracts of *A. phylicoides* were similar in terms of selected phytochemical composition, antioxidant activity and cytotoxicity [8]. In the present study therefore, the same procedure was followed for preparing the organic and aqueous extracts for comparison purposes in this study. It would be interesting to conduct further studies, including HPLC, TLC and bioautography, on the different types of aqueous extracts, namely infusions, decoctions and cold aqueous extracts.

The bioautography screen was a worthwhile bioassay that provided very useful information which has indicated the presence of several antimicrobial phytochemicals and has given justification to further study this plant species as a new antibacterial and antifungal agent. The ethanolic extract of *Athrixia phylicoides* is a complex mixture of polyphenolics, as indirectly indicated by the reddish-pink colour of bands in bioautography, together with other compounds with a range of solubility and chromatographic behaviour. Various compounds were detected that displayed a flavonoid-type substructure as evaluated from the UV spectra. These compounds could possibly be the same flavonoids as previously isolated from the ethanol extract of *A. phylicoides*, which included the known flavonoids 5-hydroxy-6,7,8,3′,4′,5′-hexamethoxyflavon-3-ol, 3-0-demethyldigicitrin, 5,6,7,8,3′,4′-hexamethoxyflavone and quercetin, or similar flavonoids [7]. To separate the compounds in the ethanol extract fully might require a more polar stationary phase, or the utilisation of a more advanced chromatographic system as well as a more comprehensive spectral library.

In this study, the organic extracts (1–4) of *A. phylicoides* all showed similar phytochemical profiles, with similar compounds showing bioactivity against the various micro-organisms. The water extract had a distinct TLC profile, with few visible compounds, with one compound near the solvent front active against *E. coli* and a different compound active against *Candida albicans*. In the CEF solvent system, which is an intermediate polarity system, the active compounds had R_f_ values greater than 0.5, indicating that the active compounds tended to be more non-polar. As the compounds detected in the HPLC analysis were of a relatively polar nature (as they did not elute early in the HPLC separation), it is possible that the active compounds (Table 4) are not the same as those identified (Table 3). This is the first report of bioautography assays of *A. phylicoides* extracts. It is now confirmed that there are several potent antibacterial and antifungal compounds in *Athrixia phylicoides* and this justifies the need for further research.

Compounds with broad spectrum antibacterial and antifungal activity, as well as those with more selective activity, were detected in the plant extracts. Gram-positive bacteria are generally more sensitive to drug action than Gram-negative bacteria [29], mainly owing to differences in their cell wall composition, and this was shown in the case of *P. aeruginosa*, which was resistant to *A. phylicoides* extracts. The selective activity, or lack of toxicity, of active compounds in tea extracts is an important aspect to investigate, particularly as the herbal tea industry is less willing to accept evidence of traditional use as an indication of safety [32].

In the current study, two complementary methodologies, namely bioautography and HPLC analysis, were used to obtain an indication of the bioactivity of different extracts and the identity of some of the chemical compounds in the same extracts. Future work should focus on isolating the bioactive compounds using bioassay-guided fractionation followed by NMR and MS analysis to identify the purified constituents. Bioautography is an extremely useful technique to guide fractionation for isolation of antibacterial and antifungal constituents from plant extracts. During the fractionation process, HPLC traces of the fractions may be compared to the bioautography chromatograms to explore correlations between bioactive compounds and the presence of HPLC peaks. Much further research needs to be conducted on the bioactivity and toxicity of compounds in *Athrixia* bush tea.

## Conclusions

In this study, a number of extracts of *Athrixia phylicoides* aerial parts were prepared using various organic solvents and water. The organic extracts (1 – 4) had more complex phytochemical profiles (Figures 2, 3, 4, Table 3) and a higher number of antimicrobial compounds (Figure 5, Table 4) than the aqueous extract (5) as demonstrated by comparing the HPLC spectra with the TLC and bioautography results. It is imperative to know more about the composition of Zulu tea extracts and how this relates to bioactivity and bioavailability, for the purpose of enhancing beneficial aspects when sourcing plant material for cultivation purposes, and for use as chemical or biological markers for standardisation.

## Abbreviations

ACN: Acetonitrile; BEA: Benzene:ethanol:ammonium hydroxide (90:10:1); CEF: Chloroform:ethyl acetate:formic acid (5:4:1); DCM: Dichloromethane; DEE: Diethyl ether; EIMS: Electron Impact ionization Mass Spectrometry; EMW: Ethyl acetate:methanol:water (40:5.4:5); EtOAc: Ethyl acetate; EtOH: Ethanol; FA: Formic acid; HPLC: High pressure liquid chromatography; MeOH: Methanol; MIC: Minimal inhibitory concentration; MS: Mass Spectrometry; NMR: Nuclear magnetic resonance; TLC: Thin layer chromatography.

## Competing interests

The authors declare that they have no competing interests.

## Authors’ contributions

LJM assisted with designing the study, performed some of the TLC and bioautography experiments, drafted sections of the manuscript and edited the final version. VPB performed most of the TLC and bioautography. MSM designed the study and wrote sections of the paper. PS and GF conducted the HPLC analysis and wrote the relevant sections of the paper. JO contributed plant material and advice in designing and performing the study, and JNE provided advice and facilities for conducting the TLC and bioautography. All authors contributed to editing the manuscript. All authors read and approved the final manuscript.

## Pre-publication history

The pre-publication history for this paper can be accessed here:

http://www.biomedcentral.com/1472-6882/13/356/prepub
